# Crystal structure of 2,6-bis­(2,5-di­meth­oxy­phen­yl)-3,5-di­methyl­piperidin-4-one

**DOI:** 10.1107/S1600536814022041

**Published:** 2014-10-15

**Authors:** Dong Ho Park, V. Ramkumar, P. Parthiban

**Affiliations:** a Department of Biomedicinal Chemistry, Inje University, Gimhae, Gyeongnam 621 749, Republic of Korea; b Department of Chemistry, IIT Madras, Chennai 600 036, TamilNadu, India; c Department of Chemistry, VEL TECH, Avadi, Chennai 600 062, India

**Keywords:** crystal structure, chair conformation, Mannich base, piperidin-4-one

## Abstract

In the title mol­ecule, C_23_H_29_NO_5_, the central piperidine ring has a chair conformation. The planes of the two benzene rings are inclined each to other at 61.7 (1)°. The crystal packing exhibits no directional inter­actions only van der Waals contacts.

## Related literature   

For the synthesis, stereochemistry and biological actions of piperidin-4-ones, see: Sahu *et al.* (2013 ▶); Parthiban *et al.* (2011 ▶). For a related crystal structure, see: Parthiban *et al.* (2008 ▶).
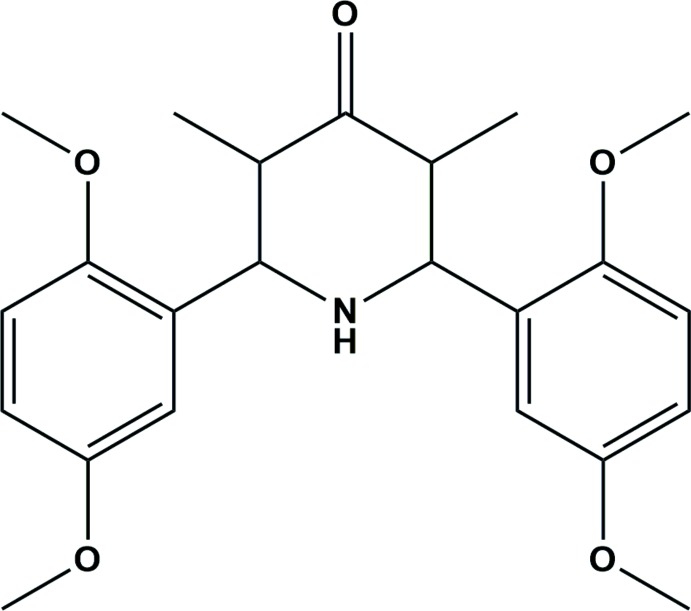


## Experimental   

### Crystal data  

C_23_H_29_NO_5_
*M* *_r_* = 399.47
Monoclinic, 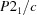

*a* = 11.1358 (7) Å
*b* = 9.4756 (5) Å
*c* = 20.4541 (11) Å
β = 92.271 (2)°
*V* = 2156.6 (2) Å^3^
*Z* = 4
Mo *K*α radiation
μ = 0.09 mm^−1^
*T* = 298 K
0.25 × 0.20 × 0.15 mm

### Data collection  

Bruker APEXII CCD area-detector diffractometer
Absorption correction: multi-scan (*SADABS*; Bruker, 2004 ▶) *T*_min_ = 0.979, *T*_max_ = 0.987
11151 measured reflections
3536 independent reflections
2262 reflections with *I* > 2σ(*I*)
*R*_int_ = 0.029

### Refinement  

*R*[*F* ^2^ > 2σ(*F* ^2^)] = 0.046
*wR*(*F* ^2^) = 0.116
*S* = 0.98
3536 reflections
272 parameters
H atoms treated by a mixture of independent and constrained refinement
Δρ_max_ = 0.17 e Å^−3^
Δρ_min_ = −0.18 e Å^−3^

### 

Data collection: *APEX2* (Bruker, 2004 ▶); cell refinement: *APEX2* and *SAINT* (Bruker, 2004 ▶); data reduction: *SAINT* and *XPREP* (Bruker, 2004 ▶); program(s) used to solve structure: *SHELXS97* (Sheldrick, 2008 ▶); program(s) used to refine structure: *SHELXL97* (Sheldrick, 2008 ▶); molecular graphics: *ORTEP-3 for Windows* (Farrugia, 2012 ▶); software used to prepare material for publication: *SHELXTL* (Sheldrick, 2008 ▶).

## Supplementary Material

Crystal structure: contains datablock(s) global, I. DOI: 10.1107/S1600536814022041/cv5470sup1.cif

Structure factors: contains datablock(s) I. DOI: 10.1107/S1600536814022041/cv5470Isup2.hkl

Supporting information file. DOI: 10.1107/S1600536814022041/cv5470Isup3.cml

**Fig. 1.**
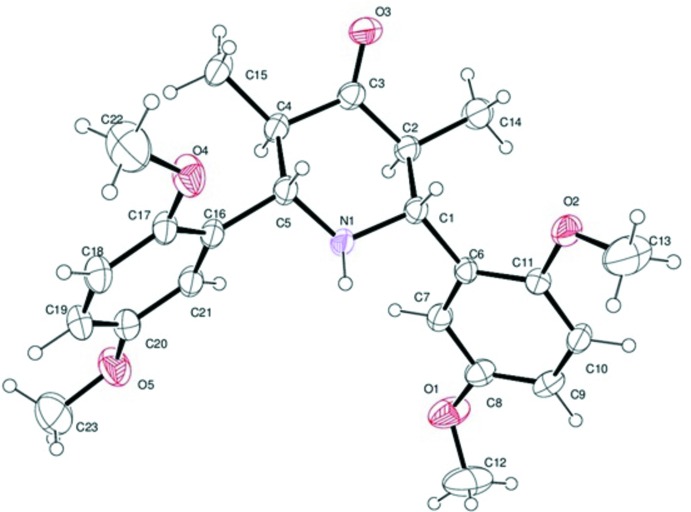
View of (I) showing the atomic numbering and 30% probability displacement ellipsoids.

. DOI: 10.1107/S1600536814022041/cv5470fig1.tif
View of (I) showing the atomic numbering and 30% probability displacement ellipsoids.

CCDC reference: 1027842

Additional supporting information:  crystallographic information; 3D view; checkCIF report

## supplementary crystallographic information

### S1. Comment

The piperidin-4-one pharmacophore is responsible for numerous biological actions such as antibacterial, antimycobacterial, antifungal, anticancer, antioxidant, antiinflammatory, neuronal nicotinistinic, and *CNS* stimulant and depressant. Its activity is further increased by the incorporation of aryl groups on both sides of the hetero atom along with/without the introduction of functionalities on the hetero atom itself. Interestingly, the amino group of the piperidone that is flanked by aryl groups are responsible not only for the increment in activity, but also in suppressing the toxicity (Sahu *et al.* 2013; Parthiban *et al.* 2011). Generally, the piperidin-4-one moiety exists in different stereochemistries upon the modifications in their structure. Since the stereochemistry of the molecule is an important key for its biological response, it is of curious to explore the stereochemistry. Hence the present study is caried out to explore the stereochemistry of the title compound (I) (Fig. 1).

The crystallographic parameters *viz*., torsion angles, asymmetry parameters and ring puckering parameters calculated for (I) show that the piperidone ring adopts a chair conformation. According to Cremer & Pople and Nardelli, the total puckering amplitude, Q_T_ is 0.5875 (8) Å, the phase angle θ is 0.94 (8)° and phi is 34 (4)°. The smallest displacement asymmetry parameters q_2_ and q_3_ are 0.0114 (8) and -0.5874 Å, respectively.

The benzene rings of anisyl groups are oriented at an angle of 61.7 (1)°, respect to each other. The torsion angles of C6—C1—C2—C3 and C3—C4—C5—C16 are 174.94 (18) and -174.42 (18)°, respectively. Similarly, the torsion angles of C2—C3—C4—C15 and C14—C2—C3—C4 are -177.8 (2) and 177.1 (2)%, respectively. The torsion angle values also clearly confirm the equatorial orientation of aryl and alkyl groups on the piperidin-4-one moiety.

On the whole, the complete crystallographic analysis of the title compound, C_23_H_29_NO_5_, exhibits a chair conformation with equatorial orientations of all the aryl and alkyl substituents.

### S2. Experimental

The 2,6-*bis*(2,5-dimethoxyphenyl)-3,5-dimethylpiperidin-4-one was synthesized by a modified and an optimized Mannich condensation in one-pot, using 2,5-dimethoxybenzaldehyde (0.1 mol, 16.618 g), 2-pentanone (0.05 mol) and ammonium acetate (0.075 mol, 5.78 g) in a 50 ml of absolute ethanol (Parthiban *et al.*, 2011). The mixture was gently warmed on a hot plate at 303–308 K (30–35° C) with moderate stirring till the complete consumption of the starting materials, which was monitored by TLC. At the end, the crude azabicyclic ketone was separated by filtration and gently washed with 1:5 cold ethanol-ether mixture. X-ray diffraction quality crystals of the title compound were obtained by slow evaporation from ethanol.

### S3. Refinement

All hydrogen atoms were fixed geometrically and allowed to ride on the parent carbon atoms with aromatic C—H = 0.93 Å, aliphatic C—H = 0.98 Å, methylene C—H = 0.97 Å. The displacement parameters were set for phenyl, methylene and aliphatic H atoms at *U*_iso_(H) = 1.2*U*_eq_(C), methyl H atoms at *U*_iso_(H) = 1.5*U*_eq_(C) and the hydrogen atoms were fixed geometrically and allowed to ride on the parent nitrogen atom with N—H = 0.86 Å and the displacement parameter was set at *U*_iso_(H)= 1.2*U*_eq_(N).

### Crystal data

**Table:**

C_23_H_29_NO_5_	*Z* = 4
*M**_r_* = 399.47	*F*(000) = 856
Monoclinic, *P*2_1_/*c*	*D*_x_ = 1.230 Mg m^−^^3^
*a* = 11.1358 (7) Å	Mo *K*α radiation, λ = 0.71073 Å
*b* = 9.4756 (5) Å	µ = 0.09 mm^−^^1^
*c* = 20.4541 (11) Å	*T* = 298 K
β = 92.271 (2)°	Block, yellow
*V* = 2156.6 (2) Å^3^	0.25 × 0.20 × 0.15 mm

### Data collection

**Table:**

Bruker APEXII CCD area-detector diffractometer	2262 reflections with *I* > 2σ(*I*)
phi and ω scans	*R*_int_ = 0.029
Absorption correction: multi-scan (*SADABS*; Bruker, 2004)	θ_max_ = 25.0°, θ_min_ = 2.4°
*T*_min_ = 0.979, *T*_max_ = 0.987	*h* = −12→12
11151 measured reflections	*k* = −11→10
3536 independent reflections	*l* = −20→24

### Refinement

**Table:**

Refinement on *F*^2^	0 restraints
Least-squares matrix: full	Hydrogen site location: mixed
*R*[*F*^2^ > 2σ(*F*^2^)] = 0.046	H atoms treated by a mixture of independent and constrained refinement
*wR*(*F*^2^) = 0.116	*w* = 1/[σ^2^(*F*_o_^2^) + (0.0405*P*)^2^ + 0.9547*P*] where *P* = (*F*_o_^2^ + 2*F*_c_^2^)/3
*S* = 0.98	(Δ/σ)_max_ < 0.001
3536 reflections	Δρ_max_ = 0.17 e Å^−^^3^
272 parameters	Δρ_min_ = −0.18 e Å^−^^3^

### Special details

**Table:**

Geometry. All e.s.d.'s (except the e.s.d. in the dihedral angle between two l.s. planes) are estimated using the full covariance matrix. The cell e.s.d.'s are taken into account individually in the estimation of e.s.d.'s in distances, angles and torsion angles; correlations between e.s.d.'s in cell parameters are only used when they are defined by crystal symmetry. An approximate (isotropic) treatment of cell e.s.d.'s is used for estimating e.s.d.'s involving l.s. planes.

### Fractional atomic coordinates and isotropic or equivalent isotropic displacement parameters (Å^2^)

**Table:**

	*x*	*y*	*z*	*U*_iso_*/*U*_eq_	
C1	0.54320 (17)	0.4952 (2)	0.14728 (9)	0.0380 (5)	
H1	0.5445	0.3918	0.1468	0.046*	
C2	0.59048 (18)	0.5485 (2)	0.21515 (10)	0.0439 (5)	
H2	0.5876	0.6519	0.2147	0.053*	
C3	0.50346 (19)	0.4971 (2)	0.26472 (10)	0.0447 (6)	
C4	0.37508 (18)	0.5434 (3)	0.25295 (10)	0.0465 (6)	
H4	0.3737	0.6469	0.2526	0.056*	
C5	0.33417 (17)	0.4916 (2)	0.18398 (9)	0.0413 (5)	
H5	0.3343	0.3882	0.1834	0.050*	
C6	0.61644 (17)	0.5504 (2)	0.09201 (9)	0.0369 (5)	
C7	0.6022 (2)	0.6895 (2)	0.07205 (10)	0.0461 (6)	
H7	0.5514	0.7484	0.0946	0.055*	
C8	0.6620 (2)	0.7426 (2)	0.01924 (11)	0.0510 (6)	
C9	0.7397 (2)	0.6581 (3)	−0.01319 (10)	0.0520 (6)	
H9	0.7807	0.6936	−0.0484	0.062*	
C10	0.7570 (2)	0.5200 (3)	0.00665 (10)	0.0495 (6)	
H10	0.8106	0.4631	−0.0150	0.059*	
C11	0.69529 (18)	0.4653 (2)	0.05833 (9)	0.0404 (5)	
C12	0.6457 (4)	0.9225 (3)	−0.06178 (14)	0.1112 (13)	
H12A	0.7285	0.9324	−0.0725	0.167*	
H12B	0.6053	1.0112	−0.0681	0.167*	
H12C	0.6080	0.8525	−0.0896	0.167*	
C13	0.7419 (4)	0.2273 (3)	0.03408 (15)	0.1146 (14)	
H13A	0.6913	0.2362	−0.0048	0.172*	
H13B	0.7329	0.1347	0.0523	0.172*	
H13C	0.8242	0.2416	0.0233	0.172*	
C14	0.7187 (2)	0.5047 (3)	0.23158 (12)	0.0684 (8)	
H14A	0.7390	0.5282	0.2763	0.103*	
H14B	0.7718	0.5533	0.2034	0.103*	
H14C	0.7266	0.4047	0.2255	0.103*	
C15	0.2921 (2)	0.4933 (3)	0.30574 (12)	0.0722 (8)	
H15A	0.2847	0.3924	0.3037	0.108*	
H15B	0.2143	0.5355	0.2988	0.108*	
H15C	0.3251	0.5203	0.3480	0.108*	
C16	0.20999 (18)	0.5443 (2)	0.16386 (10)	0.0425 (5)	
C17	0.10969 (19)	0.4552 (3)	0.16077 (11)	0.0487 (6)	
C18	0.0001 (2)	0.5069 (3)	0.13831 (12)	0.0595 (7)	
H18	−0.0658	0.4466	0.1353	0.071*	
C19	−0.0138 (2)	0.6459 (3)	0.12020 (12)	0.0610 (7)	
H19	−0.0886	0.6794	0.1055	0.073*	
C20	0.0835 (2)	0.7349 (3)	0.12398 (11)	0.0534 (6)	
C21	0.1949 (2)	0.6839 (3)	0.14593 (10)	0.0480 (6)	
H21	0.2604	0.7448	0.1486	0.058*	
C22	0.0276 (3)	0.2382 (4)	0.19599 (19)	0.1093 (13)	
H22A	−0.0150	0.2881	0.2287	0.164*	
H22B	0.0536	0.1484	0.2131	0.164*	
H22C	−0.0244	0.2240	0.1580	0.164*	
C23	−0.0296 (3)	0.9292 (4)	0.08139 (18)	0.1084 (12)	
H23A	−0.0539	0.8772	0.0428	0.163*	
H23B	−0.0200	1.0270	0.0704	0.163*	
H23C	−0.0897	0.9201	0.1135	0.163*	
N1	0.41929 (15)	0.5441 (2)	0.13674 (9)	0.0418 (5)	
O1	0.6391 (2)	0.88138 (18)	0.00307 (9)	0.0849 (6)	
O2	0.70918 (15)	0.32842 (17)	0.07975 (7)	0.0610 (5)	
O3	0.53408 (14)	0.4179 (2)	0.30899 (8)	0.0667 (5)	
O4	0.12791 (14)	0.31712 (18)	0.17881 (9)	0.0684 (5)	
O5	0.08013 (16)	0.8756 (2)	0.10695 (10)	0.0790 (6)	
H1N	0.3918 (19)	0.522 (2)	0.0956 (11)	0.054 (7)*	

### Atomic displacement parameters (Å^2^)

**Table:**

	*U*^11^	*U*^22^	*U*^33^	*U*^12^	*U*^13^	*U*^23^
C1	0.0316 (12)	0.0430 (13)	0.0398 (12)	−0.0014 (10)	0.0056 (9)	−0.0003 (9)
C2	0.0343 (13)	0.0565 (14)	0.0410 (12)	−0.0045 (11)	0.0012 (9)	0.0011 (10)
C3	0.0425 (14)	0.0560 (15)	0.0355 (12)	−0.0051 (11)	0.0007 (10)	−0.0054 (11)
C4	0.0396 (14)	0.0601 (15)	0.0405 (12)	0.0024 (11)	0.0086 (10)	−0.0017 (11)
C5	0.0325 (13)	0.0491 (14)	0.0428 (12)	−0.0005 (10)	0.0065 (9)	−0.0020 (10)
C6	0.0293 (12)	0.0436 (13)	0.0378 (11)	−0.0002 (10)	0.0020 (9)	−0.0019 (9)
C7	0.0478 (14)	0.0469 (15)	0.0442 (13)	0.0044 (11)	0.0081 (10)	−0.0026 (10)
C8	0.0621 (16)	0.0460 (15)	0.0452 (13)	−0.0051 (13)	0.0062 (12)	0.0016 (11)
C9	0.0586 (16)	0.0618 (17)	0.0364 (12)	−0.0123 (13)	0.0106 (11)	0.0012 (11)
C10	0.0435 (14)	0.0636 (17)	0.0422 (13)	0.0039 (12)	0.0122 (10)	−0.0060 (11)
C11	0.0377 (13)	0.0461 (14)	0.0374 (11)	0.0030 (11)	0.0028 (10)	−0.0005 (10)
C12	0.191 (4)	0.077 (2)	0.065 (2)	0.003 (2)	0.001 (2)	0.0232 (16)
C13	0.212 (4)	0.059 (2)	0.075 (2)	0.041 (2)	0.034 (2)	−0.0018 (16)
C14	0.0398 (15)	0.111 (2)	0.0547 (15)	−0.0071 (15)	−0.0020 (11)	0.0093 (15)
C15	0.0505 (16)	0.117 (2)	0.0500 (15)	0.0073 (16)	0.0181 (12)	0.0062 (15)
C16	0.0325 (13)	0.0549 (15)	0.0407 (12)	0.0019 (11)	0.0079 (9)	−0.0023 (10)
C17	0.0326 (14)	0.0611 (17)	0.0530 (14)	0.0022 (12)	0.0087 (10)	−0.0008 (12)
C18	0.0338 (15)	0.0736 (19)	0.0712 (17)	−0.0039 (13)	0.0049 (12)	0.0005 (14)
C19	0.0351 (15)	0.083 (2)	0.0648 (17)	0.0121 (15)	0.0022 (12)	0.0024 (14)
C20	0.0474 (16)	0.0579 (17)	0.0556 (15)	0.0122 (14)	0.0087 (12)	0.0018 (12)
C21	0.0368 (14)	0.0584 (16)	0.0494 (13)	0.0013 (12)	0.0085 (10)	−0.0027 (11)
C22	0.065 (2)	0.098 (3)	0.165 (3)	−0.0243 (19)	0.002 (2)	0.051 (2)
C23	0.096 (3)	0.102 (3)	0.128 (3)	0.043 (2)	0.012 (2)	0.030 (2)
N1	0.0293 (10)	0.0615 (13)	0.0347 (10)	0.0010 (9)	0.0031 (8)	−0.0022 (9)
O1	0.1432 (19)	0.0502 (12)	0.0627 (12)	0.0013 (11)	0.0232 (11)	0.0144 (9)
O2	0.0819 (12)	0.0503 (10)	0.0522 (10)	0.0217 (9)	0.0209 (8)	0.0043 (8)
O3	0.0544 (11)	0.0958 (14)	0.0499 (10)	0.0008 (10)	0.0020 (8)	0.0231 (9)
O4	0.0420 (10)	0.0605 (12)	0.1031 (14)	−0.0078 (9)	0.0086 (9)	0.0132 (10)
O5	0.0660 (13)	0.0680 (13)	0.1032 (15)	0.0206 (10)	0.0044 (10)	0.0128 (11)

### Geometric parameters (Å, º)

**Table:**

C1—N1	1.463 (2)	C13—O2	1.396 (3)
C1—C6	1.513 (3)	C13—H13A	0.9600
C1—C2	1.550 (3)	C13—H13B	0.9600
C1—H1	0.9800	C13—H13C	0.9600
C2—C14	1.511 (3)	C14—H14A	0.9600
C2—C3	1.511 (3)	C14—H14B	0.9600
C2—H2	0.9800	C14—H14C	0.9600
C3—O3	1.215 (2)	C15—H15A	0.9600
C3—C4	1.506 (3)	C15—H15B	0.9600
C4—C15	1.525 (3)	C15—H15C	0.9600
C4—C5	1.545 (3)	C16—C21	1.381 (3)
C4—H4	0.9800	C16—C17	1.399 (3)
C5—N1	1.467 (2)	C17—O4	1.373 (3)
C5—C16	1.512 (3)	C17—C18	1.377 (3)
C5—H5	0.9800	C18—C19	1.376 (3)
C6—C7	1.388 (3)	C18—H18	0.9300
C6—C11	1.394 (3)	C19—C20	1.372 (3)
C7—C8	1.386 (3)	C19—H19	0.9300
C7—H7	0.9300	C20—O5	1.378 (3)
C8—C9	1.369 (3)	C20—C21	1.389 (3)
C8—O1	1.378 (3)	C21—H21	0.9300
C9—C10	1.381 (3)	C22—O4	1.400 (3)
C9—H9	0.9300	C22—H22A	0.9600
C10—C11	1.384 (3)	C22—H22B	0.9600
C10—H10	0.9300	C22—H22C	0.9600
C11—O2	1.376 (2)	C23—O5	1.405 (3)
C12—O1	1.387 (3)	C23—H23A	0.9600
C12—H12A	0.9600	C23—H23B	0.9600
C12—H12B	0.9600	C23—H23C	0.9600
C12—H12C	0.9600	N1—H1N	0.91 (2)
			
N1—C1—C6	108.28 (16)	H13A—C13—H13B	109.5
N1—C1—C2	108.30 (16)	O2—C13—H13C	109.5
C6—C1—C2	112.50 (16)	H13A—C13—H13C	109.5
N1—C1—H1	109.2	H13B—C13—H13C	109.5
C6—C1—H1	109.2	C2—C14—H14A	109.5
C2—C1—H1	109.2	C2—C14—H14B	109.5
C14—C2—C3	112.79 (18)	H14A—C14—H14B	109.5
C14—C2—C1	113.20 (18)	C2—C14—H14C	109.5
C3—C2—C1	106.98 (16)	H14A—C14—H14C	109.5
C14—C2—H2	107.9	H14B—C14—H14C	109.5
C3—C2—H2	107.9	C4—C15—H15A	109.5
C1—C2—H2	107.9	C4—C15—H15B	109.5
O3—C3—C4	122.4 (2)	H15A—C15—H15B	109.5
O3—C3—C2	122.1 (2)	C4—C15—H15C	109.5
C4—C3—C2	115.40 (18)	H15A—C15—H15C	109.5
C3—C4—C15	113.20 (19)	H15B—C15—H15C	109.5
C3—C4—C5	107.28 (16)	C21—C16—C17	118.5 (2)
C15—C4—C5	112.49 (19)	C21—C16—C5	119.26 (19)
C3—C4—H4	107.9	C17—C16—C5	122.2 (2)
C15—C4—H4	107.9	O4—C17—C18	123.2 (2)
C5—C4—H4	107.9	O4—C17—C16	117.0 (2)
N1—C5—C16	108.48 (17)	C18—C17—C16	119.8 (2)
N1—C5—C4	108.59 (17)	C17—C18—C19	121.3 (2)
C16—C5—C4	112.15 (17)	C17—C18—H18	119.4
N1—C5—H5	109.2	C19—C18—H18	119.4
C16—C5—H5	109.2	C20—C19—C18	119.5 (2)
C4—C5—H5	109.2	C20—C19—H19	120.2
C7—C6—C11	118.11 (19)	C18—C19—H19	120.2
C7—C6—C1	119.29 (18)	C19—C20—O5	124.5 (2)
C11—C6—C1	122.56 (19)	C19—C20—C21	119.8 (2)
C8—C7—C6	121.4 (2)	O5—C20—C21	115.7 (2)
C8—C7—H7	119.3	C16—C21—C20	121.1 (2)
C6—C7—H7	119.3	C16—C21—H21	119.4
C9—C8—O1	123.8 (2)	C20—C21—H21	119.4
C9—C8—C7	119.9 (2)	O4—C22—H22A	109.5
O1—C8—C7	116.3 (2)	O4—C22—H22B	109.5
C8—C9—C10	119.6 (2)	H22A—C22—H22B	109.5
C8—C9—H9	120.2	O4—C22—H22C	109.5
C10—C9—H9	120.2	H22A—C22—H22C	109.5
C11—C10—C9	120.8 (2)	H22B—C22—H22C	109.5
C11—C10—H10	119.6	O5—C23—H23A	109.5
C9—C10—H10	119.6	O5—C23—H23B	109.5
O2—C11—C10	122.92 (19)	H23A—C23—H23B	109.5
O2—C11—C6	116.93 (18)	O5—C23—H23C	109.5
C10—C11—C6	120.1 (2)	H23A—C23—H23C	109.5
O1—C12—H12A	109.5	H23B—C23—H23C	109.5
O1—C12—H12B	109.5	C1—N1—C5	115.22 (16)
H12A—C12—H12B	109.5	C1—N1—H1N	110.3 (13)
O1—C12—H12C	109.5	C5—N1—H1N	109.3 (13)
H12A—C12—H12C	109.5	C8—O1—C12	118.8 (2)
H12B—C12—H12C	109.5	C11—O2—C13	117.55 (19)
O2—C13—H13A	109.5	C17—O4—C22	117.8 (2)
O2—C13—H13B	109.5	C20—O5—C23	117.3 (2)
			
N1—C1—C2—C14	−179.85 (19)	C7—C6—C11—C10	0.1 (3)
C6—C1—C2—C14	−60.2 (2)	C1—C6—C11—C10	177.74 (19)
N1—C1—C2—C3	55.3 (2)	N1—C5—C16—C21	−45.6 (3)
C6—C1—C2—C3	174.94 (18)	C4—C5—C16—C21	74.4 (2)
C14—C2—C3—O3	−6.6 (3)	N1—C5—C16—C17	132.4 (2)
C1—C2—C3—O3	118.5 (2)	C4—C5—C16—C17	−107.7 (2)
C14—C2—C3—C4	177.1 (2)	C21—C16—C17—O4	179.87 (19)
C1—C2—C3—C4	−57.8 (2)	C5—C16—C17—O4	1.9 (3)
O3—C3—C4—C15	5.9 (3)	C21—C16—C17—C18	1.9 (3)
C2—C3—C4—C15	−177.8 (2)	C5—C16—C17—C18	−176.0 (2)
O3—C3—C4—C5	−118.8 (2)	O4—C17—C18—C19	−179.4 (2)
C2—C3—C4—C5	57.5 (2)	C16—C17—C18—C19	−1.6 (4)
C3—C4—C5—N1	−54.6 (2)	C17—C18—C19—C20	0.6 (4)
C15—C4—C5—N1	−179.68 (19)	C18—C19—C20—O5	179.7 (2)
C3—C4—C5—C16	−174.42 (18)	C18—C19—C20—C21	0.1 (4)
C15—C4—C5—C16	60.5 (3)	C17—C16—C21—C20	−1.3 (3)
N1—C1—C6—C7	44.2 (2)	C5—C16—C21—C20	176.73 (19)
C2—C1—C6—C7	−75.5 (2)	C19—C20—C21—C16	0.3 (3)
N1—C1—C6—C11	−133.4 (2)	O5—C20—C21—C16	−179.4 (2)
C2—C1—C6—C11	106.9 (2)	C6—C1—N1—C5	176.09 (17)
C11—C6—C7—C8	1.5 (3)	C2—C1—N1—C5	−61.6 (2)
C1—C6—C7—C8	−176.22 (19)	C16—C5—N1—C1	−176.60 (17)
C6—C7—C8—C9	−1.9 (3)	C4—C5—N1—C1	61.3 (2)
C6—C7—C8—O1	178.8 (2)	C9—C8—O1—C12	30.9 (4)
O1—C8—C9—C10	179.9 (2)	C7—C8—O1—C12	−149.8 (3)
C7—C8—C9—C10	0.7 (3)	C10—C11—O2—C13	−28.2 (3)
C8—C9—C10—C11	0.9 (3)	C6—C11—O2—C13	153.0 (3)
C9—C10—C11—O2	179.8 (2)	C18—C17—O4—C22	−21.9 (4)
C9—C10—C11—C6	−1.3 (3)	C16—C17—O4—C22	160.3 (2)
C7—C6—C11—O2	179.04 (18)	C19—C20—O5—C23	−2.8 (4)
C1—C6—C11—O2	−3.3 (3)	C21—C20—O5—C23	176.8 (2)
