# Supplementary figures and images for: Crystal structure of 2,6-bis­(2,5-di­meth­oxy­phen­yl)-3,5-di­methyl­piperidin-4-one
Source: Acta Crystallogr Sect E Struct Rep Online. 2014 Oct 15;70(Pt 11):o1160. doi: 10.1107/S1600536814022041 (PMC4257243; doi:10.1107/S1600536814022041)

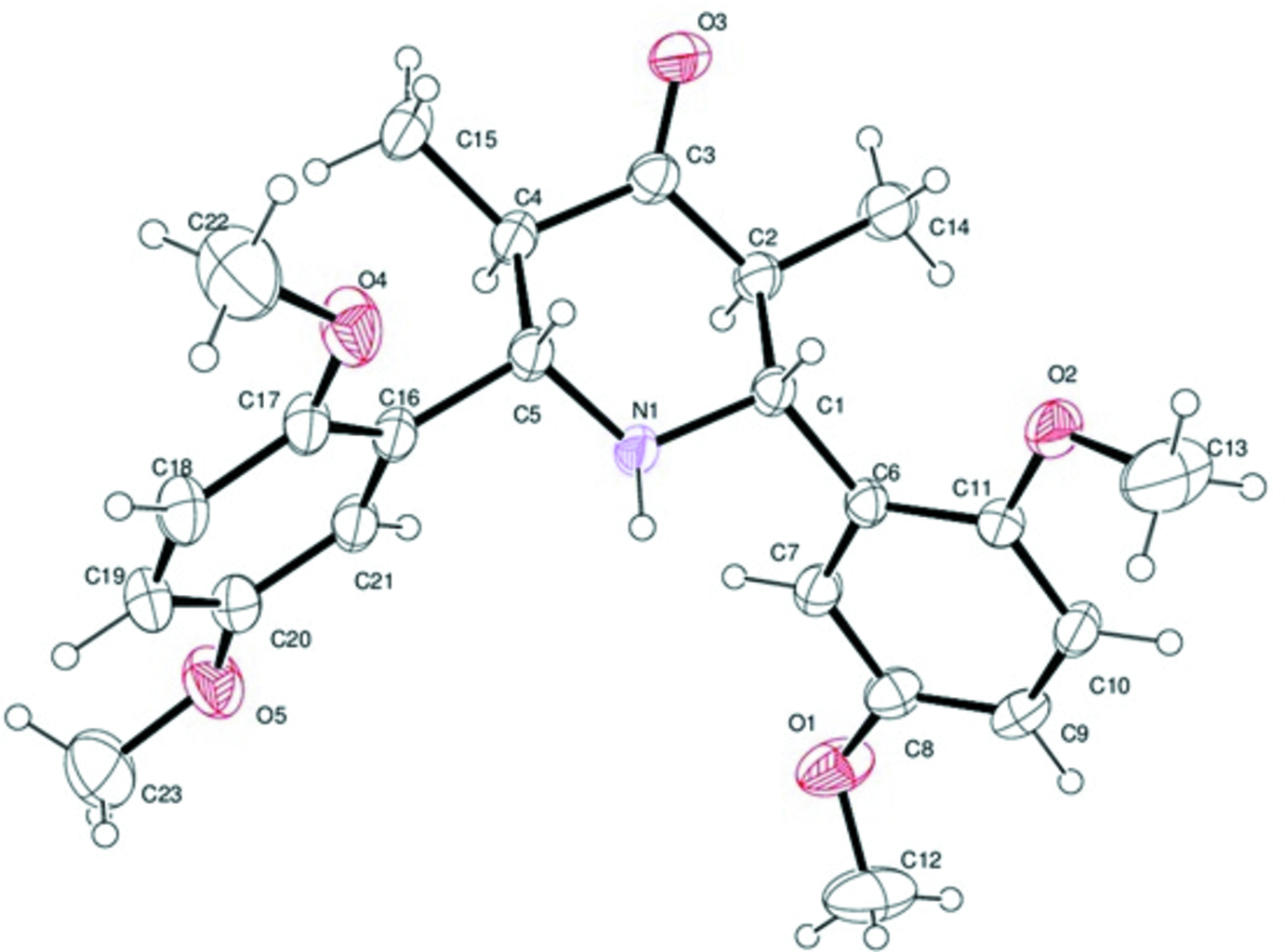

Supplement: Supplementary file 4 [file e-70-o1160-fig1.tif]
